# Bioadhesive Perivascular Microparticle-Gel Drug Delivery System for Intimal Hyperplasia Prevention: In Vitro Evaluation and Preliminary Biocompatibility Assessment

**DOI:** 10.3390/gels8120776

**Published:** 2022-11-28

**Authors:** Tamara Melnik, Alexandre Porcello, François Saucy, Florence Delie, Olivier Jordan

**Affiliations:** 1 Institute of Pharmaceutical Sciences of Western Switzerland, University of Geneva, Rue Michel-Servet 1, 1211 Geneva, Switzerland; 2 School of Pharmaceutical Sciences, University of Geneva, Rue Michel-Servet 1, 1211 Geneva, Switzerland; 3 Service of Vascular Surgery, Department of Heart and Vessels, University Hospital, Rue du Bugnon 36, 1011 Lausanne, Switzerland; 4 Service of Vascular Surgery, Ensemble Hospitalier de la Côte, Rue du Crêt 2, 1110 Morges, Switzerland

**Keywords:** intimal hyperplasia, hyaluronic acid, dopamine, mussel-inspired adhesive, perivascular application, atorvastatin

## Abstract

Intimal hyperplasia (IH) is an undesirable pathology occurring after peripheral or coronary bypass surgery. It involves the proliferation and migration of vascular smooth muscle cells, leading to a reduction in the diameter of the vascular lumen, which can lead to stenosis and graft failure. Topically applied atorvastatin (ATV) has been shown to slow down this process. To be effective, the drug delivery system should remain at the perivascular site for 5–8 weeks, corresponding to the progression of IH, and be capable of releasing an initial dose of the drug followed by a sustained release. Ideally, bioadhesion would anchor the gel to the application site. To meet these needs, we encapsulated ATV in a 2-component system: a hyaluronic acid–dopamine bioadhesive gel for rapid release and biodegradable microparticles for sustained release. The system was characterized by scanning electron microscopy, rheology, bioadhesion on porcine arteries, and a release profile. The rheological properties were adequate for perivascular application, and we demonstrated superior bioadhesion and cohesion compared to the control HA formulations. The release profile showed a burst, generated by free ATV, followed by sustained release over 8 weeks. A preliminary evaluation of subcutaneous biocompatibility in rats showed good tolerance of the gel. These results offer new perspectives on the perivascular application towards an effective solution for the prevention of IH.

## 1. Introduction

Bypass grafting is a surgical intervention performed to re-establish circulation to circumvent arteries blocked by atherosclerotic plaques. One of the most common grafts used for this purpose is the autologous saphenous vein graft [1], where a healthy vein segment is harvested from the patient. Up to 40% of vein grafts fail within years of the initial intervention [2,3]. Intimal hyperplasia (IH) is one of the leading causes of vein graft failure [3].

IH is characterized by successive hyperacute, acute, and chronic stages [4]. The hyperacute stage is triggered immediately after the surgery by the platelets accumulated at the injury site, the coagulation cascade, and the growth factors released by the platelets. Several hours into the IH progression, the acute stage begins and lasts up to several weeks post-surgery. The key players in the acute stage are the inflammation and the vascular smooth muscle cells (VSMCs). Under the influence of the growth factors and the inflammation, the VSMCs switch their phenotype and become dedifferentiated. They start to proliferate and migrate into the intimal layer, causing intimal expansion into the vessel lumen. The chronic stage can develop during the post-surgery months. It is characterized by continuous VSMC proliferation and migration, as well as matrix metalloproteinases upregulation and extracellular matrix resynthesis [4]. As a result, stenosis occurs [5], which can lead to significant lumen loss (>50%) and grave consequences, such as stroke, coronary ischemia or limb ischemia, limb loss, and the need for revascularization.

As a localized disease, IH would benefit from a local treatment. However, to this day no local formulation for the prevention of IH is available. However, our group has reported successful IH inhibition with a local DDS composed of a gel and microspheres for the controlled release of atorvastatin. This DDS has shown the potential to inhibit IH both in cell culture [6] and in animal studies [7].

Atorvastatin (ATV) is reported to act in the three stages of IH through different mechanisms. In the hyperacute stage, it promotes endothelial healing [8]. In the acute stage, it attenuates the VSMC proliferation and migration into tunica intima [9]. In the chronic stage, it downregulates matrix metalloproteinases [10]. Ideally, an effective formulation should release the ATV over the time covering these stages, and in adequate doses. Previous studies have established that for efficient IH prevention, both a burst release of a high dose in the first days and a sustained release over several weeks are required [7]. However, when scaling the system up to larger animals, this formulation needed improvements in terms of the residence time of the DDS and the ATV dose that could be loaded into the formulation [11].

Hydrogels are widely studied and used for biomedical applications [12,13]. Hyaluronic acid (HA) is often used for its biocompatibility and biodegradability, and, thanks to the various molecular weights and cross-linking possibilities, the HA-based formulations can have diverse rheological properties [14]. Depending on the method and the degree of cross-linking, HA can take the form of viscous solutions [15], mucoadhesive gels [16], and nanogels [17], as reviewed in [18]. One of the ways to cross-link HA is through catechol conjugation [19,20]. This process allows the production of the cohesive and adhesive gels that are expected to integrate with the surrounding tissues and are suitable for sustained drug delivery applications [21,22]. Due to the rapid drug release from hydrogels, the addition of drug reservoirs may be required to extend the drug release [23].

In this study, we applied a pH-triggered, HA-dopamine (HA-Dop) cross-linking method, as described previously [22], to formulate the 2-component DDS. We loaded the gel with free ATV, ATV-loaded poly(d,l-lactide-*co*-glycolide acid) (PLGA) microparticles (MPs), and a combination of both, to target the hyperacute, acute, and chronic stages of the IH development. Furthermore, we studied the incorporation of the drug and the MPs into the gel by electron microscopy; we studied the drug release kinetics, rheological properties, adhesion to vascular tissues, and the biocompatibility of the gel in vivo in rats and compared the formulations with a commercially available cross-linked HA.

## 2. Results and Discussion

### 2.1. Formulation of MPs

**Figure 1:**
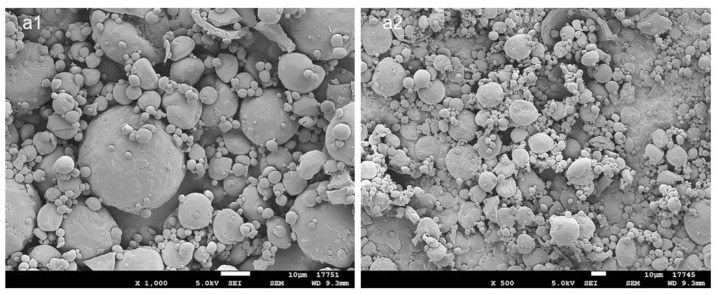
SEM images of the PLGA MPs loaded with 17.4% ATV: (**a1**) ×1000, (**a2**) ×500. Scale bar: 10 μm.

PLGA MPs with 20% nominal drug loading (DL) were prepared by spray drying. Scanning electron microscopy (SEM) images are presented in Figure 1. The MP’s size is polydisperse, as expected from the atomization process, and roughly spherical. No ATV crystals can be observed on the surface, suggesting that the drug is distributed in the polymer matrix. The mix of solvents for the spray-drying solution was selected to allow the solubilization of both the polymer and the drug: dichloromethane is a low-boiling-point solvent, typically used for PLGA solubilization, while ATV is freely soluble in methanol. The 9:1 mix of dichloromethane and methanol is azeotropic, with the mixture components having similar evaporation rates, which is favorable for homogenous drug distribution in the obtained MPs. The MPs had a high DL of 17.4% (*w*/*w*) which represents an 87% encapsulation efficiency. The diameter size was 19.5 ± 9.8 μm (D[4;3]).

### 2.2. Formulation of HA-Dop Gel

The dopamine conjugation to the HA was confirmed and quantified by ^1^H-NMR (Supplementary Figure S1). According to our previous findings, the HA-Dop formulations at the degree of substitution (DS) of 5% and 10% were bioadhesive and non-toxic towards the fibroblasts, the cells present in the adventitia of the vessels [24].

Three formulations were chosen for a demonstration before a trained vascular surgeon: HA-Dop at DS 10%, 2.0% *w*/*v*; HA-Dop at DS 10%, 1.5% *w*/*v*; and HA-Dop at DS 5%, 2.0% *w*/*v*. The surgeon handled the gels and assessed the applicability of the formulations. In parallel, oscillatory rheological measurements were performed on the formulations (Supplementary Figure S2). HA-Dop at DS 10% at 2.0% *w*/*v* concentration was considered too stiff for perivascular application (tan δ 0.2, G′ at 1 Hz: 99.5 Pa). HA-Dop DS 10% at 1.5% *w*/*v* and DS 5% at 2.0% had similar G′ and tan δ, and among those two, the candidate selected by the surgeon was HA-Dop at DS 10%, with a concentration of 1.5%.

### 2.3. Formulation of MP-Gel DDS

**Figure 2:**
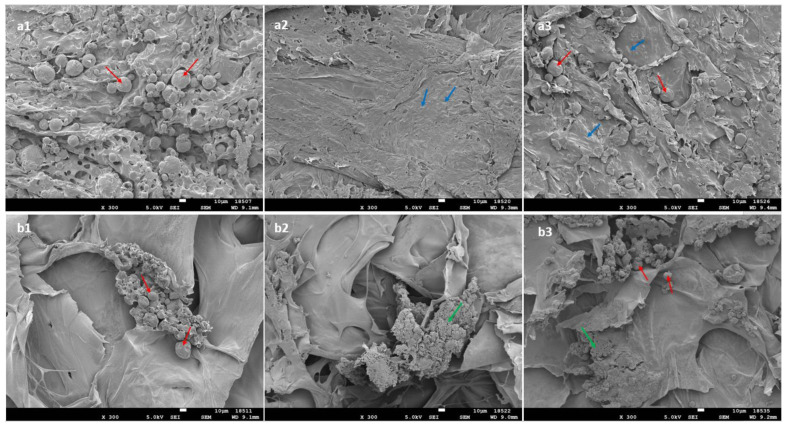
SEM images of the formulations: HA-Dop with the addition of (**a1**) MP, (**a2**) free ATV, (**a3**) MP+free ATV; Belotero Balance^®^ with the addition of (**b1**) MP, (**b2**) free ATV, (**b3**) MP+free ATV. Red arrows indicate MP; blue arrows: crystals of free ATV; green arrows: aggregates of ATV crystals. Scale bar: 10 μm; magnification: ×300.

d-α-Tocopheryl polyethylene glycol 1000 succinate (TPGS) 0.1% was used as a surfactant with a proven safety track record to help resuspend the MPs in the HA-Dop. The cross-linking was performed afterwards, providing a homogenous distribution of the powder in the gel (Figure 2). The upper panel of Figure 2 shows the homogenously distributed MPs (a1), the ATV crystals (a2), and both the MP and the ATV crystals (a3). In the lower panel of Figure 2, the MPs (b1), the ATV (b2), and the combination of both (b3) in the control gel formed clusters and were not distributed homogenously. For the commercial reference, the cross-linked gel was mixed with the drug and the particles, which generated aggregation, as is visible in the bottom panel of Figure 2.

### 2.4. Rheological and Adhesive Properties of the DDS

**Figure 3:**
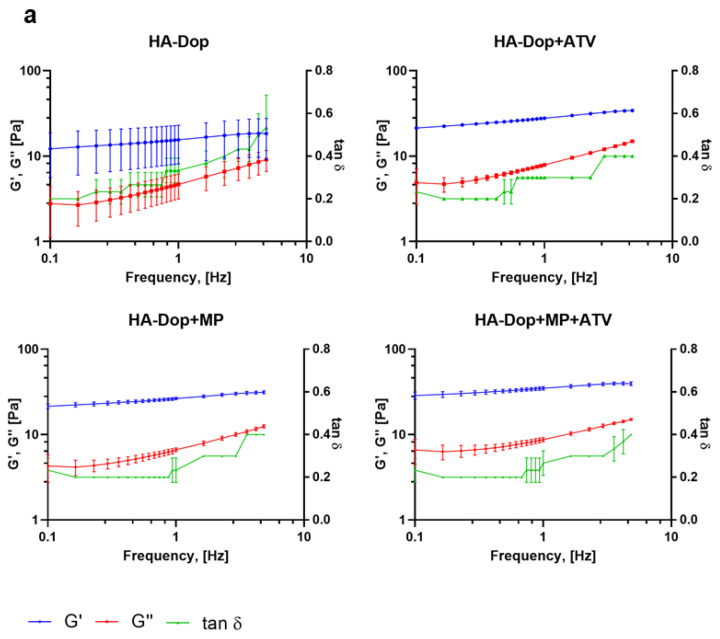

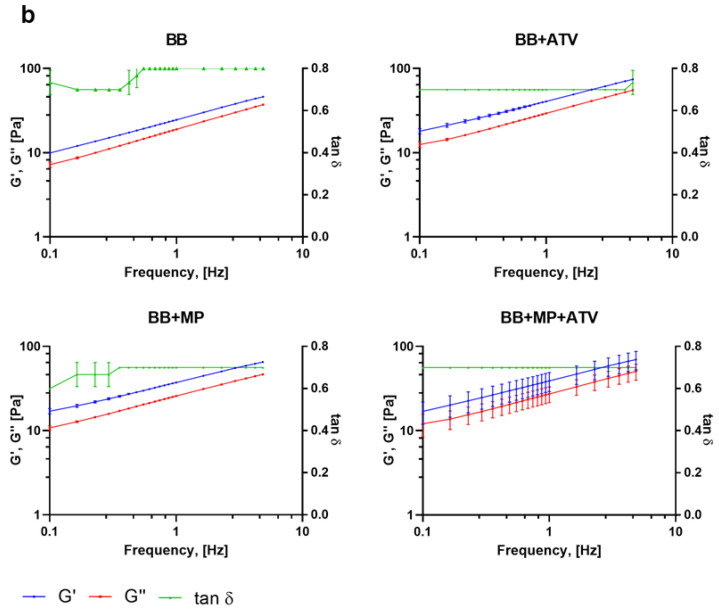
Frequency sweep of the formulations: (**a**) HA-Dop formulations, (**b**) BB formulations. N = 3. At an oscillation frequency of 1 Hz, all the gels have similar rheological properties; however, with increasing frequency, HA-Dop-based formulations remain stable throughout the sweep, while BB gels elastic modulus increases.

The oscillatory rheological measurements are presented in Figure 3.

These results show that all the formulations were gels, with the elastic modulus (G′) staying above the viscous modulus (G″) throughout the frequencies, with tan δ thus being below 1. The HA-Dop formulations had a tan δ twofold lower (0.4) compared to the BB formulations (0.8), pointing at a semisolid gel-like material. In the case of perivascular application, high frequencies are not expected, the human pulse being in the range of 0.6–2 Hz [25]. While the HA-Dop formulations were relatively stable at all the tested frequencies, the BB was showing a frequency-dependent behavior. HA-Dop alone has lower G′ values than HA-Dop with the free ATV, MPs, or both. The thickening induced by the MPs is expected for a 2.8% *v*/*v* incorporation of microspheres [26]. As for the effect of ATV, it may be that the calcium in the ATV contributed to the cross-linking through ionic mechanisms [27]. To verify this hypothesis, we compared HA-Dop gels loaded with 2.5, 5, and 10 mg/mL ATV and 0.5, 1, and 2 CaCl_2_ equivalents of 5 mg/mL ATV (in Supplementary Figures S3 and S4, the results are presented on a linear scale to more easily observe the differences). The elastic modulus was increasing with the increasing concentration of ATV, and the tan δ were similar between the gels. A similar, more pronounced trend was observed in the HA-Dop, cross-linked by CaCl_2_. The larger magnitude of this effect was due to the solubility of CaCl_2_ in water, which is around 1000 times higher than that of ATV calcium [28,29]. Overall, these results confirm the suspected dopamine–calcium interaction.

**Figure 4:**
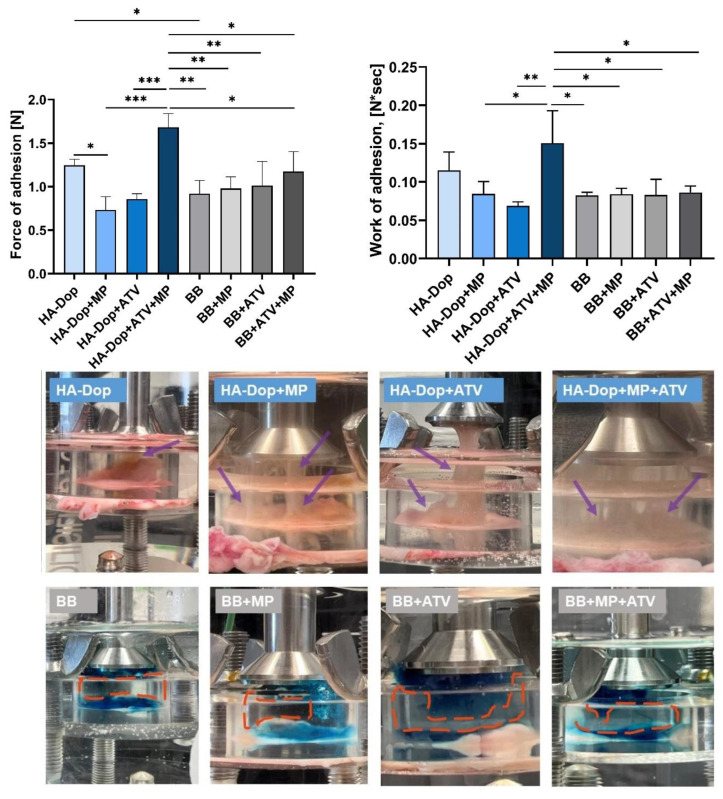
Upper panel, left: maximum of the force of adhesion [N] of the HA-Dop and control formulations, measured with texture analyzer. Right: work of adhesion [N*sec] of the formulations. All row means were analyzed by one-way ANOVA and unpaired two-tailed *t*-tests. Bottom panel: photos, taken a few seconds after the detachment of the probe. N = 3, error bars = SD, * *p* ≤ 0.05; **: *p* ≤ 0.01; ***: *p* ≤ 0.001.

Figure 4 shows that the HA-Dop alone exhibited higher adhesion force and work of the adhesion compared to the BB alone. When added to the HA-Dop individually, both the free ATV and the MPs significantly reduced the maximum adhesion force, as well as work of adhesion (not significantly). However, their addition in a combination increased both adhesion parameters. As for the BB formulations, there seemed to be no difference in the adhesion parameters whether the free ATV or MPs were added alone or in a combination. When the HA-Dop formulations are compared with the BB formulations, only the HA-Dop+ATV+MP formulation is significantly superior in adhesion compared to all the other BB formulations.

In our previous work, we compared the adhesion of HA-Dop with DS 9% to commercially available cross-linked HA and found superior adhesive properties, which was still the case in this study, even though the concentration of the gel was reduced from 2.0% to 1.5% [22]. We also modified the method of adhesion measurement to a longer contact time (20 min vs. 2 min), to allow more time for the dopamine to react with the nucleophiles (such as, −NH2, −SH) present in the biological tissues. These two modifications may have had an opposite effect—as the reduced concentration would be expected to reduce the adhesion, while the increased contact time could increase it. As a result, the adhesion trend was preserved. In the in vivo conditions, the gel would stay in contact with the vascular tissues for much longer time (several weeks); so, the 20 min contact time is still more challenging than what would happen in vivo.

The dry, insoluble MPs, as well as the poorly soluble ATV, added into the gel phase were expected to reduce adhesion by limiting molecular interactions between the gel and the tissues. This was observed when comparing the HA-Dop with the HA-Dop+ATV and HA-Dop+MP. However, in the case of HA-Dop+ATV+MP, the adhesion force was higher, although the difference was not statistically significant compared to the HA-Dop alone, but it was significantly higher than the HA-Dop+ATV and HA-Dop+MP. This could be related to the higher cohesion of this bi-component formulation due to the cross-linking reinforcement by the ATV originating from both the gel phase and the ATV released by the MPs. As established above, increasing the concentration of ATV increases G’ (Supplementary Figure S3), which provides a more cross-linked, and therefore more cohesive, gel. Additionally, the composite gel is expected to be reinforced by the hard spheres (MPs).

The differences in cohesion can also be seen in the photos of the set-up, presented in the bottom panel of Figure 4. We observed that the HA-Dop formulations were still attached to the probe and to the tissue as thick wires (purple arrows), which were still visible after the maximum adhesion force was measured, suggesting a high cohesivity of the HA-Dop formulations. The reference BB formulations showed a gap between the part of the gel that stayed on the tissue and the part that went up with the probe (orange dash-circled areas), suggesting a lower cohesion of the BB formulations.

The cohesive properties are also important for perivascular application. The formulation is ideally expected to stay as a whole, and it is likely to be degraded by the enzymes more slowly than a less cohesive formulation [30].

Bioadhesion testing is a challenging task as many factors must be taken into account. The texture analyzer mucoadhesion rig is occasionally used to determine the adhesion of polymers to biological tissues [31,32]; however, this method has some limitations. As demonstrated by the photos taken after the probe retracted and the measurements of the maximum adhesion force and work of adhesion were recorded, the HA-Dop formulations remained adherent both to the tissues and to the probe, with thick wires connecting both pieces (purple arrows). This effect was not measured as the probe did not actually tear the gel away from the tissue. Moreover, we could observe some gel remaining on the tissue after the measurement in all the eight cases. In case of the BB formulations, there were no thick wires, but the gel was torn apart, and part of it remained attached to the tissue; the detachment of the gel from the tissue did not take place; so, it was not measured. Therefore, this method of bioadhesion testing does not take all these occurrences into account and cannot accurately measure the adhesion of such viscoelastic and highly deformable materials. An accurate measurement of bioadhesion, considering all these factors, remains to be developed, and to this day, it is not uncommon to accompany the values obtained by adhesion testing machines with photos and videos demonstrating adhesion for a qualitative, visual assessment [20,33].

### 2.5. Drug Release Profile

**Figure 5:**
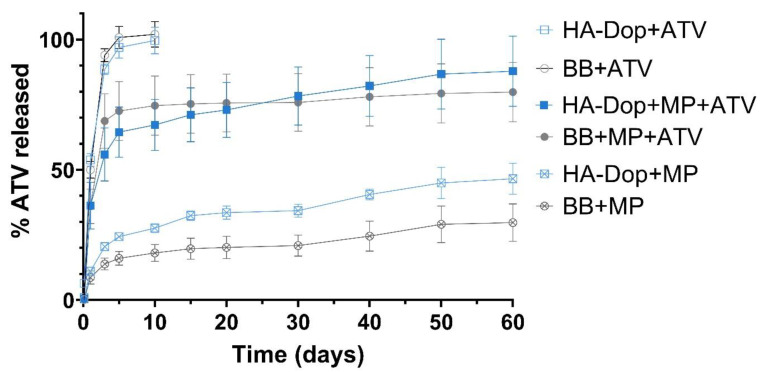
Release curves of the formulations obtained with the Transwell^®^ set-up. Free ATV is diffused within the first 10 days from both the HA-Dop and the control. In the formulations with both free ATV and MPs, there is a high burst effect, mainly provided by the free drug. In the formulations with MPs only, the release is sustained throughout the study. N = 3, error bars = SD.

The drug release profiles from the gel with free ATV, the gel with MPs, and the gel with both free ATV and ATV-loaded MPs are presented in Figure 5.

The six curves show three release profile types. The HA-Dop and BB formulations with free ATV present a high and rapid burst, which reached 100% of the total drug by day 5. The HA-Dop and BB formulations containing the free ATV in gel phase and the ATV-loaded MPs showed a burst, reaching around 50% by day 5 and continuing to release ATV until day 60. Finally, the HA-Dop and BB formulations containing only the ATV-loaded MPs did not show a clear burst and released the ATV in a sustained way over 60 days. The extraction of ATV from the remaining gel and MP matrix was performed at day 60. It showed the mass and % of total ATV still present in the formulations at the study endpoint (Supplementary Table S1). The curves, presented in Figure 5, were normalized to the total mass of the drug released plus the mass of the drug extracted at the end of the release study.

The release of the free drug was quite similar between the HA-Dop and the control, the non-functionalized HA. The same was observed with the release of ATV from the formulations containing both ATV in the gel phase and in the MPs and the formulations containing only ATV-loaded MPs. These results suggest that the presence of dopamine in the gels does not significantly impact the release profile.

The MPs showed a sustained release of ca. 40% of their payload over the investigated period. This would allow for an extended treatment over several months, although the release kinetics would certainly be accelerated in vivo in the presence of enzymatic activity. The choice of a polymer with a low molecular weight (14 kDa), a 75:25 lactide:glycolide ratio, and an ester termination was made seeking to achieve this drug release profile. The microparticles prepared with the same protocol with the polymer at 50:50 lactide:glycolide (RG 502) had a more accelerated release profile and were not selected for further experiments (data not shown).

One factor which may slow down the release rate could be the presence of drug and/or particle aggregates in the BB control formulations, as observed in Figure 2. This seems to have been the case for the formulations containing MPs but not as much for the free ATV in the gel phase, where the release profiles between HA-Dop and BB are very similar. The delayed release patterns from the aggregates of drug and MPs were avoided thanks to the oxidizer-free cross-linking process, which was performed after the addition of the drug and MPs into the gel.

Another factor to consider is the release set-up used. The Transwell^®^ system is a convenient way to keep the formulations separated from the receptor wells. This sampling procedure decreases the risk of sampling the formulation together with the release medium or the risk of otherwise disturbing the formulation. However, it may have artificially slowed down the release. We compared the release of ATV from the same MPs alone, either in flasks with the MPs or in the Transwell^®^ system. The results suggest that the Transwell^®^ system indeed sustained the release in addition to the effect exerted by the polymer matrix of the MPs (Supplementary Figure S5). This effect may be attributed to the smaller radius of the Transwell^®^ inserts, providing milder agitation, and to the immobilization of the MPs in the small-radius inserts, while the MPs could float more freely in the Erlenmeyer flasks.

In summary, the proposed bi-component formulation possesses a release profile suitable to target the hyper-acute phase of the IH, with the help of the burst ATV released in the hyperacute stage of IH and the remaining ATV released in a sustained way, to address the progression of the disease into the acute and chronic stages.

### 2.6. Subcutaneous Implantation in Rats for Biocompatibility Testing

**Figure 6:**
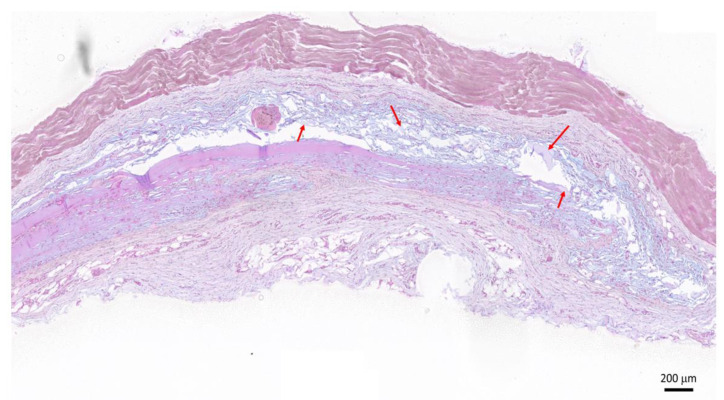
Histological section of the HA-Dop implant area 28 days after implantation, hematoxylin/eosin and alcian blue staining. Red arrows indicate the blue-stained HA residues. Scale bar: 200 μm.

After the subcutaneous implantation of the HA-Dop and BB gels, the animals exhibited normal food intake, water consumption, and behavior during the study (28 days). Five out of ten injected HA-Dop gels could be retrieved on the histological sections. The blue-stained HA gel remnants (red arrows) indicated degradation of the gel (Figure 6). The histopathological analysis revealed no significant inflammatory reaction, as confirmed by the absence of fibrosis, macrophages, or polymorphonuclear leukocytes around the gel remnants. Neither acute neutrophilic nor giant cell reaction was observed.

This indicated a good tolerability of the gels. The relatively fast degradation was expected in the rat subcutaneous compartment, which was prone to gel leaking between fasciae. Indeed, similar degradation was observed with the cross-linked commercial formulations designed for skin repair (Supplementary Figure S6). Further validation in a large animal model of IH, using MP-loaded formulations, is warranted to assess the effective performances of a drug-loaded delivery system.

## 3. Conclusions

Our aim was to formulate a perivascular DDS with the specific features needed for efficient IH prevention. The rheological properties of the DDS should allow practitioners to easily apply the gel to the vessel anastomoses. The adhesion and cohesion of the gel should comply with a residence time of 5 to 8 weeks in vivo until full drug release and degradation. The system should also provide a possibility to deliver high doses of drugs into the gel phase, as well as into the sustained release reservoirs, to allow high burst release, followed by a sustained release up to 8 weeks.

A composite gel was designed with bi-compartment ATV loading to reach the desired biphasic release: a burst release, followed by a sustained release phase. A drug load as high as 10 mg/mL was incorporated into the system; the high ATV payload in the MPs allowed the incorporation of a high dose of ATV while maintaining the targeted rheological and adhesive properties of the DDS. The oxidizer-free cross-linking process, performed after the addition of the drug and the MPs allowed their homogenous distribution and avoided spontaneous release patterns generated by the drug and particle aggregates. The dopamine functionalization of HA increased the bioadhesion of the bi-compartment DDS, which is expected to ensure its prolonged residence at the anastomose site. Moreover, the presence of dopamine did not have a major influence on the release kinetics of the drug out of the DDS, nor on the biocompatibility of the gel, as confirmed in vivo. Altogether, our findings indicate an optimal profile of the HA-Dop based perivascular bi-compartment DDS for further use in animal studies and subsequent use in the clinics.

## 4. Materials and Methods

### 4.1. Materials

PLGA (Resomer RG752s, MW 10–20 kDa) was purchased from Evonik (Essen, Germany). Atorvastatin calcium was obtained from Chemos GmbH&Co.KG (Altdorf, Germany). The dichloromethane, methanol, ethanol, and acetonitrile were HPLC grade and purchased from Fisher Scientific (Loughborough, UK).

HA (molecular weight range 130–300 kDa) was purchased from Contipro Biotech Ltd., Dolní Dobrouč, Czech Republic. Dopamine hydrochloride, phosphate buffer saline (PBS), sodium hydroxide (NaOH), hydrochloric acid (HCl), (N-(3-dimethylaminopropyl)-N′-ethylcarbodiimide hydrochloride (EDC), N-hydroxysulfosuccinimide sodium (sNHS), TPGS, sodium dodecyl sulfate (SDS), and D_2_O were purchased from Sigma-Aldrich. The anhydrous calcium chloride and alcian blue were from HÄNSELER AG, Germany. Sterile 0.9% NaCl was obtained from B.Braun (Merlsungen, Germany). Cross-linked HA, Belotero Balance^®^ (BB) was purchased from Merz Pharma (Allschwil, Switzerland) and served as a reference as unmodified HA gel.

### 4.2. Formulation of ATV-Loaded MPs by Spray Drying

PLGA MPs were produced with a 4 M8-TriX spray dryer (ProCepT, Zelzate, Belgium). Briefly, atorvastatin was added into the PLGA solution in dichloromethane:methanol (9:1) at a 20% *w*/*w* concentration (% of total dry ingredients). Ten milliliters of the solution was then sprayed through the spray dryer’s nozzle at a set feed rate. The following parameters were used (Table 1):

The MPs were collected into an air-tight vial and stored at 4 °C until further use.

### 4.3. Characterization of MP Size and Drug Loading

Particle size was determined by laser light diffraction using a Mastersizer S (Malvern Panalytical, Malvern, UK). Approximately 1 mg of MPs was suspended in 1 mL of 0.1% TPGS and placed in an ultrasonic bath for 5 min. The result is the averaged record of 5 measurements, obtained in Malvern Mastersizer software. The refractive index was set at 1.59 for MPs and 1.33 for water.

To measure drug loading, approximately 3 mg of MPs was solubilized in 10 mL of the mixture of acetonitrile:ethanol (1:1). After 4 h agitation on a magnetic stirrer, the solution was diluted 4× in the mobile phase (60% *v*/*v* aqueous solution of formic acid (0.1% *v*/*v*) and 10 mM ammonium formate, 40% *v*/*v* acetonitrile with 0.1% formic acid). The samples were analyzed using a Waters Acquity™ Ultraperformance LC (Milford, MA, USA) equipped with an Acquity UPLC^®^ BEH C18 column (2.1 mm × 50 mm, 1.7 µm, Waters, USA) equilibrated at 30 °C. The calibration curve was made by dissolving ATV in an acetonitrile/ethanol (1:1) solution, containing 0.1 mg/mL PLGA, in the concentration range from 0.78 to 25 μg/mL ATV. Eluates were monitored at 245 nm, retention time: 2.1 min, software used for data collection and treatment was: Empower 2, Waters Corporation, Milford, MA, USA. The drug loading results are presented as the percent mass ratio of the encapsulated drug to the total mass of the MPs.

### 4.4. Synthesis of HA-Dop

HA-Dop with a DS of dopamine relative to HA dimeric units (DS) of 5 and 10% was synthesized as previously described [22]. The molecular weight of HA in the range of 130–300 kDa was selected based on previous reports on catechol conjugation to HA [22,34,35]. Briefly, to obtain the HA-Dop with a DS of 10%, 250 mg of HA was solubilized in 25 mL dH2O under magnetic stirring, followed by the addition of 3 HA-COO- equivalents of EDC and 1.3 sNHS equivalents. Thirty minutes later, 1.3 equivalents of dopamine were added and left overnight to react. The reaction was maintained at pH 5.5 by adding NaOH or HCl dropwise. Dialysis over 48 h against PBS at pH 6 and dH2O was used to purify the HA-Dop conjugates from the unreacted components. After dialysis, the mixture was freeze-dried and stored at +4 °C until further use. To obtain sterile HA-Dop for in vivo implantation in rats, the same procedure was followed, and the obtained conjugate was passed through a 0.2 μm filter (Millipore Sigma, Burlington, MA, USA) before freeze-drying. To obtain HA-Dop with DS 5%, the same procedure was followed, with 2, 1.2, and 1.2 equivalents of EDC, sNHS, and dopamine, respectively.

### 4.5. Determination of the DS by ^1^H NMR

The DS was determined by ^1^H NMR on a Bruker Avance Neo 600 MHz NMR spectrometer (Bruker BioSpin, Rheinstetten, Germany) supplied with a QCI 5 mm Cryoprobe and a SampleJet automated sample changer. Freeze-dried HA-Dop samples were solubilized in D_2_O at 5 mg/mL under stirring for 2 h. The spectra were treated with MestReNova (Mnova) software V10.0 (Mestrelab, Santiago de Compostela, Spain). The D_2_O solvent peak was used as a reference peak, and the chemical shifts were reported as parts per million (ppm). DS was calculated from the ratio of the peaks of the three dopamine aromatic protons at δ = 6.7–7.0 ppm to the peak of the HA N-acetyl group (three protons) at δ = 1.9–2.0 ppm.

### 4.6. Cross-Linking of HA-Dop Gels

For cross-linking with NaOH, 15 mg of HA-Dop was solubilized in 1 mL of 0.1% TPGS aqueous solution under magnetic stirring for 3 h. When a homogenous gel was obtained, the pH was adjusted to 8.5 with 0.5 M NaOH and left to cross-link for 48 h, protected from light.

For cross-linking with CaCl_2_, 15 mg of HA-Dop was solubilized in 1 mL of 0.1% TPGS solution with CaCl_2_ concentrations of 0.25, 0.5, and 1 mg/mL on a magnetic stirrer for 3 h. Once the homogenous gel obtained, the pH was adjusted to 8.5 with 0.5 M NaOH and left to cross-link for 48 h, protected from light.

### 4.7. Loading of MPs and/or ATV into the Gels

To prepare the MP-loaded gel (HA-Dop+MP) at the dose of ATV 5 mg/mL, the MPs were suspended in 0.1% TPGS aqueous solution at 2.8% (*w*/*v*). HA-Dop was added to the solution at a 1.5% *w*/*v* concentration and left to swell and mix on a magnetic stirring plate for 3 h. After solubilization of the components, the HA-Dop was cross-linked by the addition of 0.5 M NaOH to the pH of 8.5 and left to cross-link for 48 h, protected from light.

To prepare HA-Dop+ATV at the dose of ATV 5 mg/mL, the procedure described above was followed, but free ATV was added to 0.1% TPGS at 0.5% (*w*/*v*) instead of the MPs.

For HA-Dop+MP+ATV, the procedures described above were followed, with both MP and free ATV suspended in the 0.1% TPGS solution, to have the total drug concentration of 10 mg/mL.

To mix the commercially available cross-linked HA with ATV and MP at the same doses, the powders were mixed with an already cross-linked formulation. As this gel was already cross-linked, a manual mixing was performed to avoid diluting the gels. 

Table 2 gives the concentrations of ATV in the gel phase, MP phase, as well as total ATV concentration in all the formulations.

### 4.8. SEM

To observe the formulations by SEM, they were frozen at −80 °C and freeze-dried with Christ Alpha 2–4 LD plus freeze-dryer (Kuehner AG, Birsfelden, Switzerland). The obtained sponge-like structures were thinly cut with a scalpel and placed on stubs for the SEM. They were then coated with a 15–20 nm layer of gold and examined by SEM using a JSM-7001FA (JEOL, Tokyo, Japan) at 5.0 kV.

### 4.9. Rheological Measurements

The oscillatory rheological behaviors of the formulations were determined with a HAAKE Mars Rheometer™ (ThermoFisher Scientific, Waltham, MA, USA) with a cone-plate C35 2°/Ti rotor. Approximately 400 μL of the formulations was loaded on the measuring plate, and the storage modulus (G′) and loss modulus (G″) were recorded at 25 °C, at a frequency sweep of 0.1 to 4.68 Hz. Tan δ was calculated as the ratio of G″ to G′. Each measurement was performed in triplicate.

### 4.10. Adhesion Measurements by Texture Analyzer

Porcine aortas were purchased from a local slaughterhouse, stored at 5 °C, and used within 72 h. The assay was performed using the “Gel Mucoadhesion Probe” of the Texture Analyser TA.XTPlusC (Stable Microsystems Ltd., Surrey, UK). The aortas were cut into squares of approximately 8 cm^2^. A sample was placed into the mucoadhesion rig and conditioned in a beaker of PBS, at 37 °C. Then, the probe carrying the gel (300 μL) was brought into contact with the aorta and a force of 0.5 N was applied for 20 min; the detachment speed was 10 mm/s. Three repetitions were performed for each test. For the adhesion measurements, all the BB-based reference formulations were colored with methylene blue.

### 4.11. Drug Release Profile

One hundred microliters of the formulations at 1.5% of HA-Dop, prepared as described in 4.7, was placed in 12-well plate Transwell^®^ inserts with a 0.4 μM cutoff (Corning Incorporated, #3470). Release medium (3.5 mL PBS with 0.1% SDS *w*/*v*) was added into the receiver compartment, ensuring sink conditions. The plate was placed into a rotary shaker, maintained at 37 °C, at 80 rpm. At predetermined time intervals, all the release medium was collected and weighed to determine the exact volume sampled. Three point five milliliters of fresh medium was put into the wells. The samples were then analyzed with U-HPLC, as described in 4.3. At day 60, the release medium was removed and analyzed, and the remaining gels were collected and frozen at −80 °C and freeze-dried. After freeze-drying, the gels were weighed, and a mixture of ACN:EtOH (1:1) was added to dissolve the PLGA and to extract the remaining ATV in the formulations. The samples were diluted in a mobile phase and injected into U-HPLC, as described in Section 4.3. The calibration curve was constructed with consecutive dilutions of ATV in 30% ethanol in PBS/SDS 0.1%, containing 0.01% TPGS (*w*/*v*) (ATV concentrations: 0.78–25.0 μg/mL). The release curves were constructed as the percentage of the total mass of ATV, which was released, plus the mass of ATV, extracted at the end of the release study with ACN:EtOH.

We further compared the Transwell^®^ vs. Erlenmeyer flasks release set-ups with MPs only. For the Transwell^®^, 2.0 mg of the MPs was weighed and placed into the Transwell^®^s in triplicates, and 3.5 mL of the release medium was added into the bottom wells. In a parallel experiment, 8.0 mg of the MPs was weighed into Erlenmeyer flasks in triplicates, and 15 mL of the release medium was added on top. The plate with the Transwell^®^s, as well as the flasks, was incubated in one rotary shaker maintained at 37 °C and 80 rpm. For the Transwell^®^ experiments, the sampling was conducted in the same way, as described above for the Gel+MP release study. For the MPs, incubated in solution, the MPs were allowed to sediment in static conditions; then, 500 μL of the supernatant was withdrawn from the top of the solution and replaced with fresh medium. The samples were then injected into U-HPLC and analyzed with the method described in Section 4.3. The calibration curve was constructed with consecutive dilutions of ATV in 30% ethanol in PBS/SDS 0.1% (0.78–25.0 μg/mL). Each experiment was conducted in triplicate, and the values are presented as the mean ± standard deviation.

### 4.12. Subcutaneous Implantation in Rats for Biocompatibility Testing

The rat experiments were conducted under the authorization number 34193/GE152 from the Direction Générale de la Santé Genève, according to Swiss animal law regulations. Five male Sprague Dawley rats weighing approx. 200 g were identified and kept in separate cages. They received standard food and water ad libitum. The rats were anesthetized with 2% isoflurane, and 0.3 mL of sterile gel material was injected subcutaneously, using a syringe with a 23-gauge needle, on both flank sides. For the implantation, the HA-Dop gel was prepared from pre-filtered HA-Dop, as per 2.6, with the exception that 0.9% NaCl was used instead of 0.1% TPGS solution; the preparation was performed under laminar flow in aseptic conditions. The control formulation (BB) was used as a reference. The animals were sacrificed at day 28 by intraperitoneal injection of pentobarbital. The implant area was carefully dissected, the explanted material was fixed in 4% formaldehyde, and histological slides were stained with hematoxylin/eosin to stain the tissues and with alcian blue to stain the HA.

### 4.13. Statistical Analyses

Statistical analyses were performed in GraphPad Prism 8. For the bioadhesion results, one-way ANOVA and unpaired two-tailed *t*-tests were performed to compare all the row means and the pairs of HA-Dop and BB with the respective additives.

## Figures and Tables

**Table 1:** Process parameters used for the preparation of ATV-loaded MP by spray-drying process.

Name	Value
Solvent	dichloromethane:methanol (9:1)
Batch size, [mL]	10
Polymer concentration [%, *w*/*v*]	7
DL [%, weight of total dry weight]	20
Inlet air temperature, [°C]	65
Inlet air flow [m^3^/min]	0.3
Feed rate [mL/min]	1.8
Cyclone size	Medium
Nozzle size, [mm]	0.6

**Table 2:** Summary of the investigated formulations with their drug contents. A similar labelling is used for the reference gel, where “BB” is used instead of “HA-Dop”.

Formulation	ATV in Gel Phase [mg/mL]	ATV in MP Phase [mg/mL]	Total ATV [mg/mL]
HA-Dop	0	0 (no MP)	0
HA-Dop+MP	0	5	5
HA-Dop+ATV	5	0 (no MP)	5
HA-Dop+MP+ATV	5	5	10
BB	0	0 (no MP)	0
BB+MP	0	5	5
BB+ATV	5	0 (no MP)	5
BB+ATV+MP	5	5	10

## Data Availability

The data presented in this study are available on request from the corresponding author.

## Supplementary Materials

The following supporting information can be downloaded at: https://www.mdpi.com/article/10.3390/gels8120776/s1. Figure S1: The degree of substitution (DS) of dopamine as quantified by ^1^H-NMR; Figure S2: Rheological properties of the formulations: 2% HA-Dop DS5+MP+ATV; 1.5% HA-Dop DS10+MP+ATV; 2% HA-Dop DS10+MP+ATV; Figure S3: Frequency sweep evaluation of HA-Dop alone, HA-Dop+ATV at concentrations from 2.5 to 10 mg/mL; Figure S4: Frequency sweep evaluation of HA-Dop alone, HA-Dop cross-linked with CaCl_2_ at 0.5, 1 and 2 equivalents of 5 mg/mL ATV (actual CaCl_2_ concentrations: 0.25; 0.5; 1 mg/mL); Figure S5: Comparison of the release rate of ATV from the microparticles either in an Erlenmeyer flask (MP20 sol) and in Transwell® system (MP20 TW); Figure S6: Histological section of the HA-Dop implant area 28 days after subcutaneous implantation of the BB control gel, hematoxylin/eosin and Alcian Blue staining. Table S1: Mass balance to account the ATV released and extracted from the remaining gel during the release study.

## References

[1] Hiramoto J.S., Teraa M., De Borst G.J., Conte M.S. (2018). Interventions for lower extremity peripheral artery disease. Nat. Rev. Cardiol..

[2] Almasri J., Adusumalli J., Asi N., Lakis S., Alsawas M., Prokop L.J., Bradbury A., Kolh P., Conte M.S., Murad M.H. (2019). A systematic review and meta-analysis of revascularization outcomes of infrainguinal chronic limb-threatening ischemia. J. Vasc. Surg..

[3] Owens C.D., Gasper W.J., Rahman A.S., Conte M.S. (2013). Vein graft failure. J. Vasc. Surg..

[4] Scharn D., Daamen W.F., van Kuppevelt T., van der Vliet J. (2012). Biological Mechanisms Influencing Prosthetic Bypass Graft Patency: Possible Targets for Modern Graft Design. Eur. J. Vasc. Endovasc. Surg..

[5] Elmore J.B., Mehanna E., Parikh S.A., Zidar D.A., Parikh S. (2016). Restenosis of the Coronary Arteries. Coronary and Endovascular Stents, An Issue of Interventional Cardiology Clinics.

[6] Dubuis C., May L., Alonso F., Luca L., Mylonaki I., Meda P., Delie F., Jordan O., Déglise S., Corpataux J.-M. (2013). Atorvastatin-Loaded Hydrogel Affects the Smooth Muscle Cells of Human Veins. J. Pharmacol. Exp. Ther..

[7] Mylonaki I., Strano F., Deglise S., Allémann E., Alonso F., Corpataux J.-M., Dubuis C., Haefliger J.-A., Jordan O., Saucy F. (2016). Perivascular sustained release of atorvastatin from a hydrogel-microparticle delivery system decreases intimal hyperplasia. J. Control. Release.

[8] Laufs U. (2003). Beyond lipid-lowering: Effects of statins on endothelial nitric oxide. Eur. J. Clin. Pharmacol..

[9] Chen S., Liu B., Kong D., Li S., Li C., Wang H., Sun Y. (2015). Atorvastatin Calcium Inhibits Phenotypic Modulation of PDGF-BB-Induced VSMCs via Down-Regulation the Akt Signaling Pathway. PLoS ONE.

[10] Schweitzer M., Mitmaker B., Obrand D., Sheiner N., Abraham C., Dostanic S., Meilleur M., Sugahara T., Chalifour L.E. (2009). Atorvastatin Modulates Matrix Metalloproteinase Expression, Activity, and Signaling in Abdominal Aortic Aneurysms. Vasc. Endovasc. Surg..

[11] Mylonaki I., Allain E., Strano F., Allémann E., Corpataux J.-M., Meda P., Jordan O., Delie F., Rougemont A.-L., Haefliger J.-A. (2018). Evaluating intimal hyperplasia under clinical conditions. Interact. Cardiovasc. Thorac. Surg..

[12] Hoffman A.S. (2012). Hydrogels for biomedical applications. Adv. Drug Deliv. Rev..

[13] Lin W., Kluzek M., Iuster N., Shimoni E., Kampf N., Goldberg R., Klein J. (2020). Cartilage-inspired, lipid-based boundary-lubricated hydrogels. Science.

[14] Trombino S., Servidio C., Curcio F., Cassano R. (2019). Strategies for Hyaluronic Acid-Based Hydrogel Design in Drug Delivery. Pharmaceutics.

[15] Zhang X., Wei D., Xu Y., Zhu Q. (2021). Hyaluronic acid in ocular drug delivery. Carbohydr. Polym..

[16] Kafedjiiski K., Jetti R.K.R., Föger F., Hoyer H., Werle M., Hoffer M., Bernkop-Schnürch A. (2007). Synthesis and in vitro evaluation of thiolated hyaluronic acid for mucoadhesive drug delivery. Int. J. Pharm..

[17] Pedrosa S.S., Gonçalves C., David L., Gama M. (2014). A Novel Crosslinked Hyaluronic Acid Nanogel for Drug Delivery. Macromol. Biosci..

[18] Tripodo G., Trapani A., Torre M.L., Giammona G., Trapani G., Mandracchia D. (2015). Hyaluronic acid and its derivatives in drug delivery and imaging: Recent advances and challenges. Eur. J. Pharm. Biopharm..

[19] Zhou D., Li S., Pei M., Yang H., Gu S., Tao Y., Ye D., Zhou Y., Xu W., Xiao P. (2020). Dopamine-Modified Hyaluronic Acid Hydrogel Adhesives with Fast-Forming and High Tissue Adhesion. ACS Appl. Mater. Interfaces.

[20] Kim J., Lee C., Ryu J.H. (2020). Adhesive Catechol-Conjugated Hyaluronic Acid for Biomedical Applications: A Mini Review. Appl. Sci..

[21] Hong S., Yang K., Kang B., Lee C., Song I.T., Byun E., Park K.I., Cho S.-W., Lee H. (2013). Hyaluronic Acid Catechol: A Biopolymer Exhibiting a pH-Dependent Adhesive or Cohesive Property for Human Neural Stem Cell Engineering. Adv. Funct. Mater..

[22] Melnik T., Ben Ameur S., Kanfar N., Vinet L., Delie F., Jordan O. (2022). Bioadhesive Hyaluronic Acid/Dopamine Hydrogels for Vascular Applications Prepared by Initiator-Free Crosslinking. Int. J. Mol. Sci..

[23] Garbayo E., Ruiz-Villalba A., Hernandez S., Saludas L., Abizanda G., Pelacho B., Roncal C., Sanchez B., Palacios I., Prósper F. (2021). Delivery of cardiovascular progenitors with biomimetic microcarriers reduces adverse ventricular remodeling in a rat model of chronic myocardial infarction. Acta Biomater..

[24] De Vries M.R., Simons K.H., Jukema J.W., Braun J., Quax P. (2016). Vein graft failure: From pathophysiology to clinical outcomes. Nat. Rev. Cardiol..

[25] Chang H., Chen J., Liu Y. (2018). Micro-piezoelectric pulse diagnoser and frequency domain analysis of human pulse signals. J. Tradit. Chin. Med. Sci..

[26] Zhu J., Abeykoon C., Karim N. (2021). Investigation into the effects of fillers in polymer processing. Int. J. Light. Mater. Manuf..

[27] Ryu J., Ku S.H., Lee H., Park C.B. (2010). Mussel-Inspired Polydopamine Coating as a Universal Route to Hydroxyapatite Crystallization. Adv. Funct. Mater..

[28] Ropp R.C., Ropp R.C. (2013). Chapter 2—Group 17 (H, F, Cl, Br, I) Alkaline Earth Compounds. Encyclopedia of the Alkaline Earth Compounds.

[29] Wei J., Chen S., Fu H., Wang X., Li H., Lin J., Xu F., He C., Liang X., Tang H. (2021). Measurement and correlation of solubility data for atorvastatin calcium in pure and binary solvent systems from 293.15 K to 328.15 K. J. Mol. Liq..

[30] Sall I., Férard G. (2007). Comparison of the sensitivity of 11 crosslinked hyaluronic acid gels to bovine testis hyaluronidase. Polym. Degrad. Stab..

[31] Avachat A.M., Gujar K.N., Wagh K.V. (2013). Development and evaluation of tamarind seed xyloglucan-based mucoadhesive buccal films of rizatriptan benzoate. Carbohydr. Polym..

[32] Soe M.T., Chitropas P., Pongjanyakul T., Limpongsa E., Jaipakdee N. (2019). Thai glutinous rice starch modified by ball milling and its application as a mucoadhesive polymer. Carbohydr. Polym..

[33] Di X., Hang C., Xu Y., Ma Q., Li F., Sun P., Wu G. (2019). Bioinspired tough, conductive hydrogels with thermally reversible adhesiveness based on nanoclay confined NIPAM polymerization and a dopamine modified polypeptide. Mater. Chem. Front..

[34] Shin J., Lee J.S., Lee C., Park H.-J., Yang K., Jin Y., Ryu J.H., Hong K.S., Moon S.-H., Chung H.-M. (2015). Tissue Adhesive Catechol-Modified Hyaluronic Acid Hydrogel for Effective, Minimally Invasive Cell Therapy. Adv. Funct. Mater..

[35] Zhang K., Wei Z., Xu X., Feng Q., Xu J., Bian L. (2019). Efficient catechol functionalization of biopolymeric hydrogels for effective multiscale bioadhesion. Mater. Sci. Eng. C.

