# Supplementary material for: Bioadhesive Perivascular Microparticle-Gel Drug Delivery System for Intimal Hyperplasia Prevention: In Vitro Evaluation and Preliminary Biocompatibility Assessment
Source: Gels. 2022 Nov 28;8(12):776. doi: 10.3390/gels8120776 (PMC9778534; doi:10.3390/gels8120776)
Supplement: Supplementary file 1 [file gels-08-00776-s001.zip › gels-2035965-supplementary.pdf]

Bioadhesive Perivascular Microparticle-Gel Drug Delivery System for Intimal Hyperplasia Prevention: In Vitro Evaluation and Preliminary Biocompatibility Assessment

Tamara Melnik, Alexandre Porcello, François Saucy, Florence Delie, Olivier Jordan

Supplementary materials.

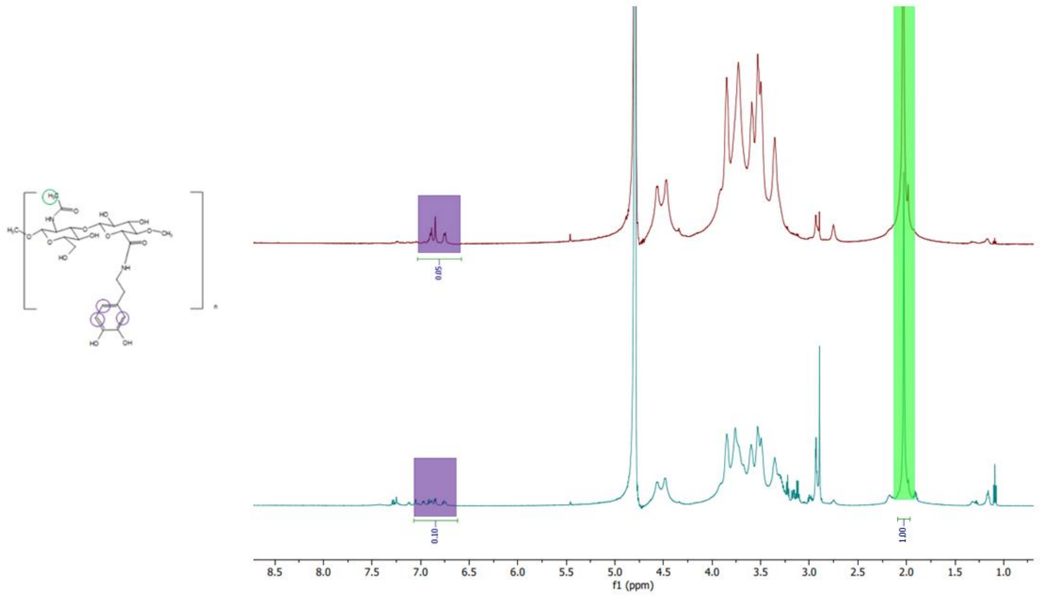

Figure S1. The degree of substitution (DS) of dopamine as quantified by ^{1}H-NMR. The acetyl peak of HA is outlined in green, the aromatic protons of dopamine – in purple. To determine the DS, the 3 protons of the acetyl peak are integrated as 1.0, and the 3 aromatic protons are integrated as a per cent of those.

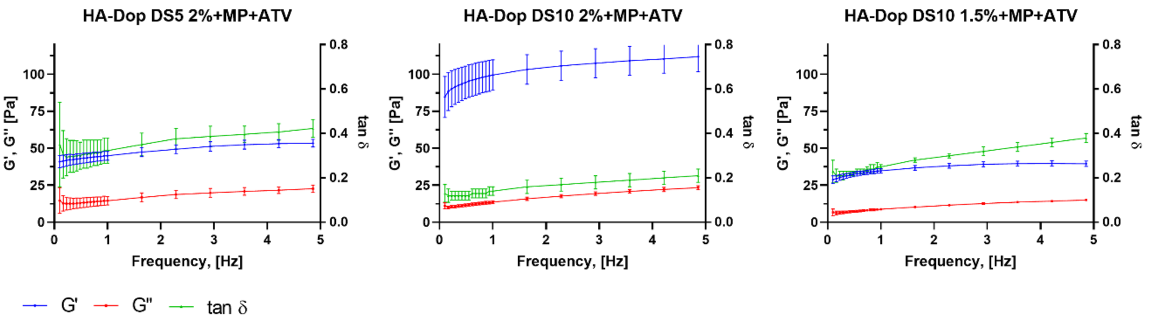

Figure S2. Rheological properties of the formulations: 2% HA-Dop DS5+MP+ATV; 1.5% HA-Dop DS10+MP+ATV; 2% HA-Dop DS10+MP+ATV.

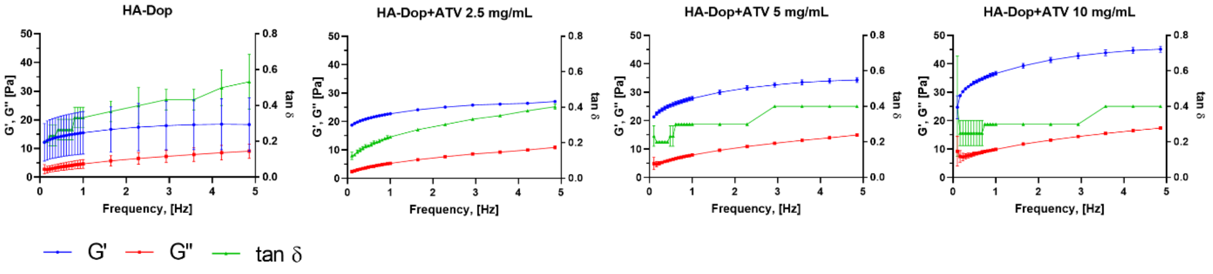

Figure S3. Frequency sweep evaluation of HA-Dop alone, HA-Dop+ATV at concentrations from 2.5 to 10 mg/mL.

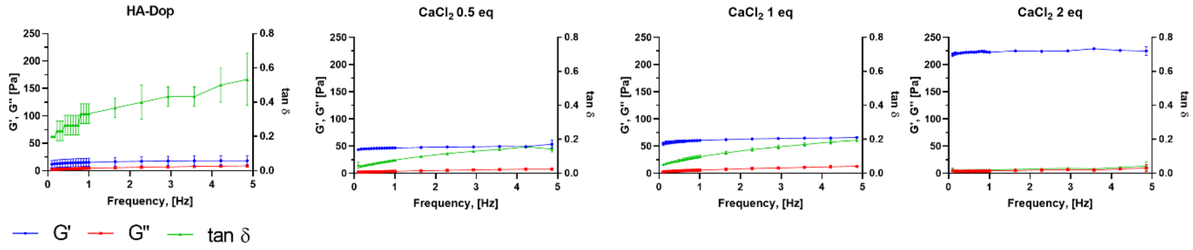

Figure S4. Frequency sweep evaluation of HA-Dop alone, HA-Dop cross-linked with $CaCl_2$ at 0.5, 1 and 2 equivalents of 5 mg/mL ATV (actual $CaCl_2$ concentrations: 0.25; 0.5; 1 mg/mL).

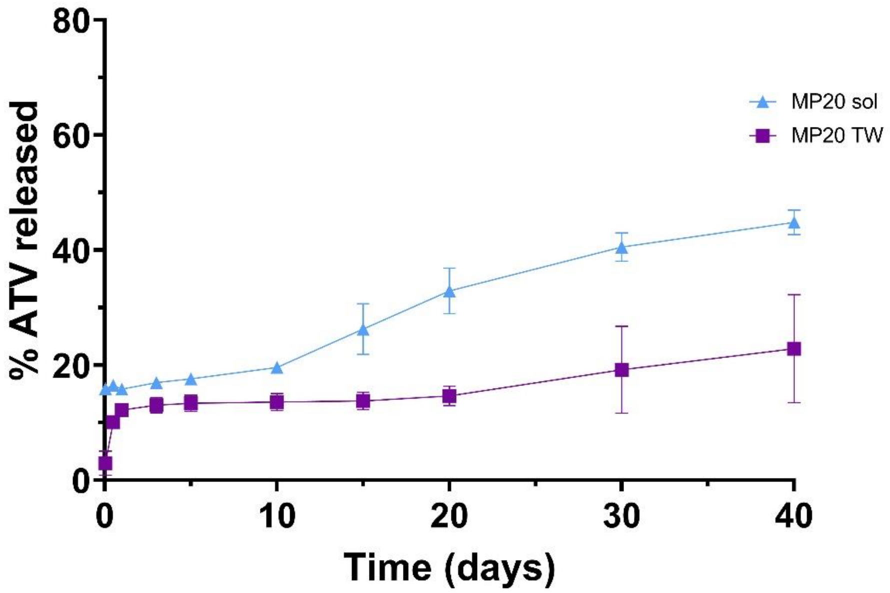

Figure S5. Comparison of the release rate of ATV from the microparticles either in an Erlenmeyer flask (MP20 sol) or in Transwell® system (MP20 TW).

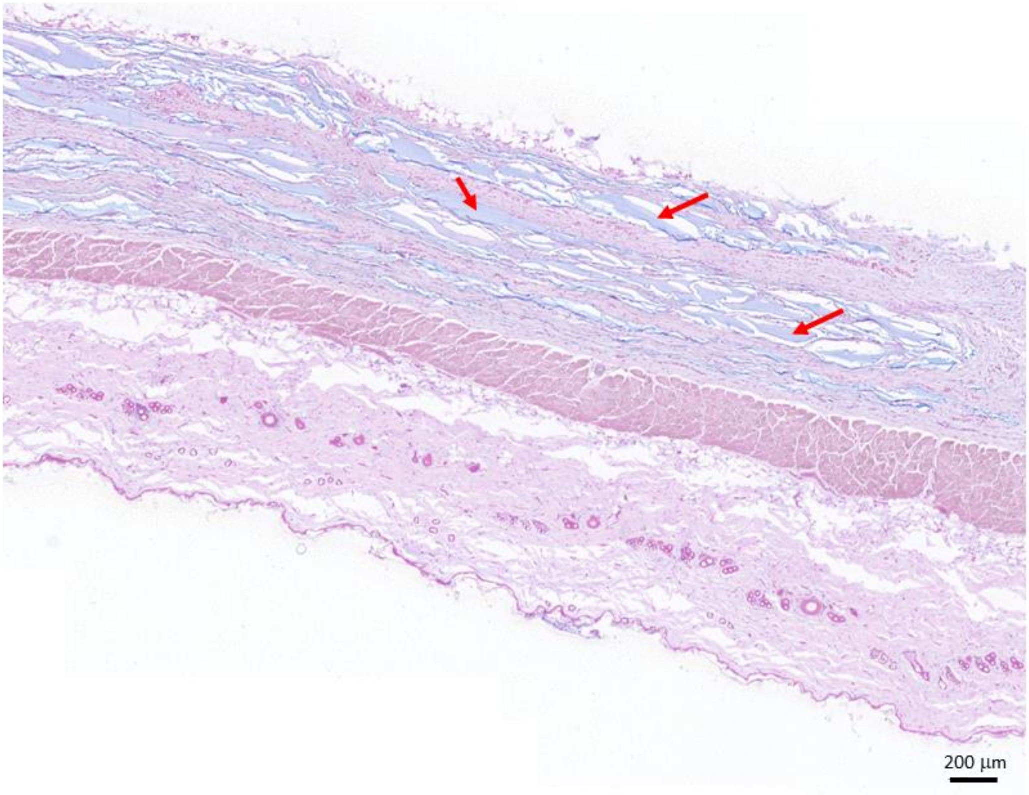

Figure S6. Histological section of the HA-Dop implant area 28 days after subcutaneous implantation of the BB control gel, hematoxylin/eosin and Alcian Blue staining. Red arrows indicate the blue-stained HA residues. Scale bar: 200 µm.

Table S1. Mass balance to account the ATV released and extracted from the remaining gel during the release study, n=3

	Total mass ATV in 100 uL of the formulation, μg	Total mass ATV released, μg	Total mass ATV extracted after the release study	Total mass released+extracted, μg	% ATV released+ extracted
HA-Dop+ATV	500	317±16	27±7	343±10	69±2
BB+ATV	500	324±11	30±9	354±7	71±1
HA-Dop+MP	500	148±19	169±11	317±21	63±4
BB+MP	500	100±24	236±112	335±133	67±27
HA-Dop+MP+ATV	1000	617±95	85±6	702±89	70±9
BB+MP+ATV	1000	554±79	140±78	694±157	69±16
